# β-Hydroxybutyric Sodium Salt Inhibition of Growth Hormone and Prolactin Secretion via the cAMP/PKA/CREB and AMPK Signaling Pathways in Dairy Cow Anterior Pituitary Cells

**DOI:** 10.3390/ijms16024265

**Published:** 2015-02-16

**Authors:** Shou-Peng Fu, Wei Wang, Bing-Run Liu, Huan-Min Yang, Hong Ji, Zhan-Qing Yang, Bin Guo, Ju-Xiong Liu, Jian-Fa Wang

**Affiliations:** 1 College of Veterinary Medicine, Jilin University, Changchun 130062, China; E-Mails: shoupengfu@163.com (S.-P.F.); wang_wei99@jlu.edu.cn (W.W.); lhglbr@163.com (B.-R.L.); yangzhanqing1983@163.com (Z.-Q.Y.); guobin79@jlu.edu.cn (B.G.); 2 College of Animal Science and Veterinary Medicine, Heilongjiang Bayi Agricultural University, Daqing 163319, China; E-Mails: yanghuanmin@aliyun.com (H.-M.Y.); jihonghljbynd@aliyun.com (H.J.)

**Keywords:** β-hydroxybutyric acid, dairy cow anterior pituitary cells, growth hormone, prolactin

## Abstract

β-hydroxybutyric acid (BHBA) regulates the synthesis and secretion of growth hormone (GH) and prolactin (PRL), but its mechanism is unknown. In this study, we detected the effects of BHBA on the activities of G protein signaling pathways, AMPK-α activity, *GH*, and *PRL* gene transcription, and GH and PRL secretion in dairy cow anterior pituitary cells (DCAPCs). The results showed that BHBA decreased intracellular cAMP levels and a subsequent reduction in protein kinase A (PKA) activity. Inhibition of PKA activity reduced cAMP response element-binding protein (CREB) phosphorylation, thereby inhibiting GH and PRL transcription and secretion. The effects of BHBA were attenuated by a specific G_αi_ inhibitor, pertussis toxin (PTX). In addition, intracellular BHBA uptake mediated by monocarboxylate transporter 1 (MCT1) could trigger AMPK signaling and result in the decrease in *GH* and *PRL* mRNA translation in DCAPCs cultured under low-glucose and non-glucose condition when compared with the high-glucose group. This study identifies a biochemical mechanism for the regulatory action of BHBA on *GH* and *PRL* gene transcription, translation, and secretion in DCAPCs, which may be one of the factors that regulate pituitary function during the transition period in dairy cows.

## 1. Introduction

Ketosis is a metabolic disorder that usually occurs in dairy cattle during the early lactation period when cows experience a state of negative energy balance and low blood glucose concentrations [1]. This disorder is characterized by elevated concentrations of the ketone bodies β-hydroxybutyrate acid (BHBA), acetoacetate and acetone in blood, urine, and milk. In animals, the pituitary is a critical regulator of a broad range of physiological processes involved in growth, metabolism, reproduction, lactation, and stress [2]. The secretion of growth hormone (GH) and prolactin (PRL) from the pituitary gland is modulated by various metabolic influences; however, *in vivo* experiments have revealed very large differences in the effect of BHBA on GH and PRL secretion depending on the physiological state of the animal. For example, Meier *et al.* found that during periods of negative energy balance, the somatotrophic axis responds by increasing plasma GH and decreasing plasma IGF-I levels [3]. Laeger *et al.* reported that high blood BHBA concentrations inhibit the secretion of GH in humans and rhesus monkeys [4]. Thus, results obtained from the studies that used different background animals are not precise. Therefore, the effect and detailed mechanisms by which BHBA mediates bovine pituitary function remain to be elucidated through *in vitro* studies.

GPR109A is a seven-transmembrane G protein-coupled receptor (GPR) of the G_αi_ family that is expressed mainly in the white adipocytes and immune cells, such as monocytes and neutrophils of humans and mice [5]. The mRNA and protein for GPR109A were observed in fat, muscle, liver and brain of Holstein steers [6]. Moreover, BHBA has been identified as an endogenous ligand of GPR109A [5]. Previously, we found that short-chain fatty acids could inhibit bovine *GH* and *PRL* gene transcription via the cAMP-PKA-CREB signaling pathway through GPR41 and GPR43 activation [7]. Thus, we hypothesize that BHBA may mediate bovine *GH* and *PRL* gene transcription via the G protein signaling pathway. AMP-activated protein kinase (AMPK) has emerged as a key molecular player in energy homeostasis at both the cellular and whole-body levels. The incubation of GT1-7 cells with BHBA in the 5.5 mM glucose medium was found to modulate AMPK-α phosphorylation in GT1-7 cells [8]. Pelletier and Coderre also found that BHBA inhibited the activation of the AMPK/p38 MAPK signaling pathway in cardiomyocytes [9]. Recent studies reported that AMPK plays a role in regulating somatotroph function both in the normal rat pituitary and in the proliferation of pituitary adenomatous cells [10]. Therefore, BHBA may regulate bovine pituitary function through the activation of AMPK-α signaling during period of low blood glucose concentrations.

The aim of the present study was to investigate the effect and mechanisms of BHBA on GH and PRL secretion in dairy cow anterior pituitary cells (DCAPCs). To achieve this aim, the activities of G protein signaling pathways, AMPK-α activity, *GH* and *PRL* gene transcription, and GH and PRL secretion were all determined. The results of this study could provide new knowledge regarding the potential effects of BHBA on bovine pituitary function.

## 2. Results

### 2.1. Effect of BHBA on mRNA Levels of GH, PRL and Pit-1 in DCAPCs

**Figure 1:**
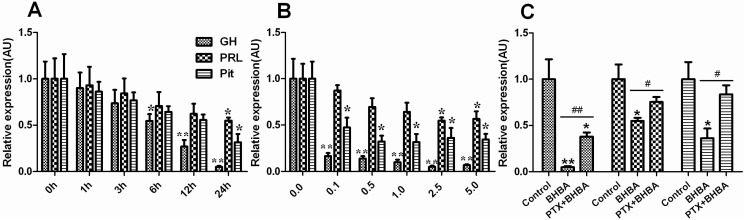
The effect of β-hydroxybutyric acid (BHBA) on mRNA levels of *GH*, *PRL* and *Pit-1* in dairy cow anterior pituitary cells (DCAPCs). (**A**) The effects of the duration of BHBA treatment on *GH*, *PRL*, and *Pit*-*1* gene expression; (**B**) The effects of the dosage of BHBA treatment on the *GH*, *PRL*, and *Pit-1* gene expression; (**C**) The results of the mRNA levels of *GH*, *PRL* and *Pit-1* in DCAPCs treated with or without prior pertussis toxin (PTX) incubation for 2 h and then stimulated with BHBA for 24 h. * indicates *p* < 0.05 *vs.* the control group, ** indicates *p* < 0.01 *vs.* the control group, **^#^** indicates *p* < 0.05 *vs.* the prior PTX incubation group, **^##^** indicates *p* < 0.01 *vs.* the prior PTX incubation group.

The mRNA levels of *GH*, *PRL*, and pituitary-specific transcription factor-1 (*Pit-1*) showed a decreasing trend in the BHBA-treated groups. The mRNA levels of *GH* were markedly lower after the 24 h BHBA treatment (Figure 1A; *p* < 0.01), and the mRNA levels of *PRL* and *Pit-1* were significantly lower after the 24 h BHBA treatment (Figure 1A; *p* < 0.05). The mRNA levels of *GH* were markedly lower in the 0.1, 0.5, 1.0, 2.5, and 5.0 mmol/L BHBA treatment groups after 24 h (Figure 1B; *p* < 0.01), the mRNA levels of *PRL* were significantly lower in the 2.5 and 5.0 mmol/L BHBA treatment groups after 24 h (Figure 1B; *p* < 05), and the mRNA levels of *Pit-1* were significantly lower in the 0.1, 0.5, 1.0, 2.5, and 5.0 mmol/L BHBA treatment groups after 24 h (Figure 1B; *p* < 0.05). The mRNA levels of *GH* were markedly higher in the PTX + BHBA group than in the BHBA treatment group (Figure 1C; *p* < 0.01), and the mRNA levels of *PRL* and *Pit-1* were significantly higher in the PTX + BHBA group than in the BHBA treatment group (Figure 1C; *p* < 0.05).

### 2.2. Effect of BHBA on GH and PRL Secretion in DCAPCs

**Figure 2:**
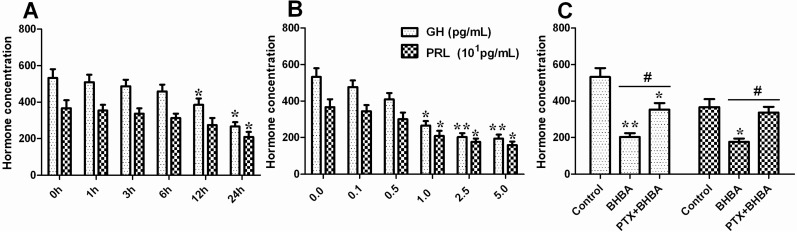
The effect of BHBA on the secretion of GH and PRL in DCAPCs. (**A**) The effects of the duration of BHBA treatment on GH and PRL secretion; (**B**) The effects of the dosage of BHBA treatment on GH and PRL secretion; (**C**) The results of the secretion levels of GH and PRL in DCAPCs treated with or without prior PTX incubation for 2 h and then stimulated with BHBA for 24 h. * indicates *p* < 0.05 *vs.* the control group, ** indicates *p* < 0.01 *vs.* the control group, **^#^** indicates *p* < 0.05 *vs.* the prior PTX incubation group.

As shown in Figure 2, BHBA notably decreased GH and PRL secretion in DCAPCs in a dose- and time-dependent manner (Figure 2A,B). The secretion levels of GH and PRL were significantly increased in the PTX + BHBA group compared with the BHBA treatment group (Figure 2C; *p* < 0.05). These results indicate that BHBA can decrease GH and PRL transcription and translation in DCAPCs.

### 2.3. Effect of BHBA on Intracellular cAMP Concentration

**Figure 3:**
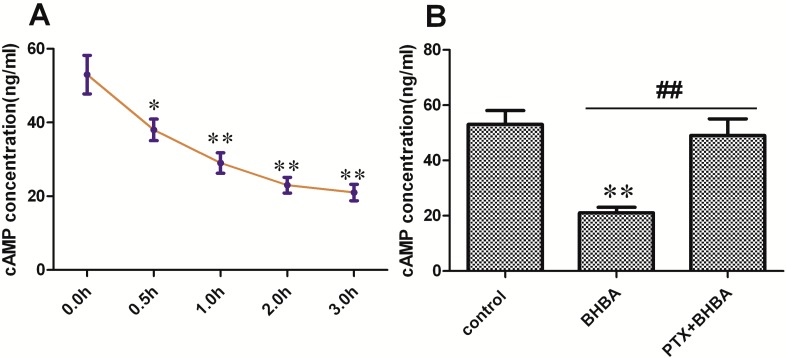
The effect of BHBA on intracellular cAMP levels in DCAPCs. (**A**) DCAPCs were treated with 2.5 mmol/L BHBA for 0, 0.5, 1.0, 2.0, and 3.0 h; (**B**) The cells were also treated with or without prior PTX incubation for 2 h and then stimulated with 2.5 mmol/L BHBA for 3 h. * indicates *p* < 0.05 *vs.* the control group, ** indicates *p* < 0.01 *vs.* the control group, **^##^** indicates *p* < 0.01 *vs.* the prior PTX incubation group.

As shown in Figure 3A BHBA notably decreased the cAMP level in DCAPCs in a time-dependent manner. The cAMP levels were markedly higher in the PTX + BHBA group than in the non-PTX treatment group (Figure 3B; *p* < 0.01). These results indicate that BHBA can decrease the intracellular cAMP concentration by activating the G_αi_ subunit in DCAPCs.

### 2.4. Effect of BHBA on PKA Activity

**Figure 4:**
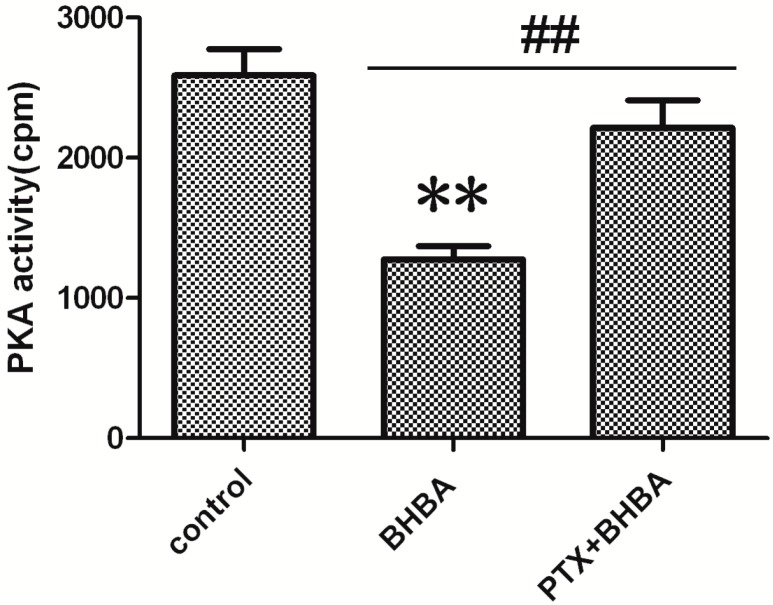
Effect of The effect of BHBA on the activity of PKA in DCAPCs. ** indicates *p* < 0.01 *vs.* the control group, **^##^** indicates *p* < 0.01 *vs.* the prior PTX incubation group.

The PKA activity was lower in the BHBA-treated groups than in the control group, and the inhibiting effect of BHBA was blocked by PTX (Figure 4). Thus, BHBA can inhibit PKA activity by decreasing the intracellular cAMP concentration in DCAPCs.

### 2.5. Effect of BHBA on CREB Phosphorylation

**Figure 5:**
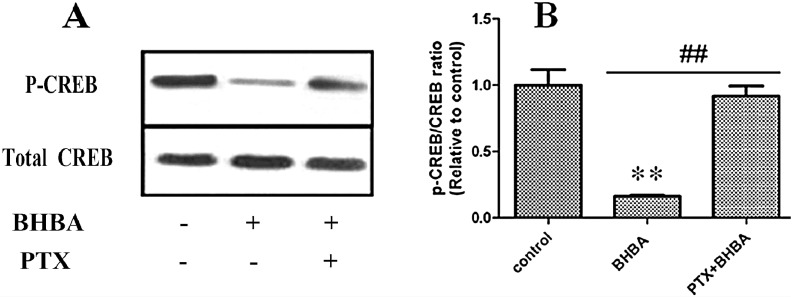
The effect of BHBA on CREB phosphorylation in DCAPCs. (**A**) The Western blotting results of p-CREB and CREB; (**B**) The phosphorylation level of CREB. ** indicates *p* < 0.01 *vs.* the control group, **^##^** indicates *p* < 0.01 *vs.* the prior PTX incubation group.

The phosphorylation levels of CREB (Figure 5) were markedly lower in the BHBA-treated group than in the control group (Figure 5A; *p* < 0.01), and the phosphorylation levels of CREB were markedly higher with prior PTX treatment (Figure 5B; *p* < 0.01). Taken together, these findings suggest that BHBA inhibit the cAMP/PKA/CREB signaling pathway to modulate *GH* and *PRL* gene transcription in DCAPCs.

### 2.6. Effect of BHBA on the mRNA Levels of GPR109A and MCT1 in DCAPCs

**Figure 6:**
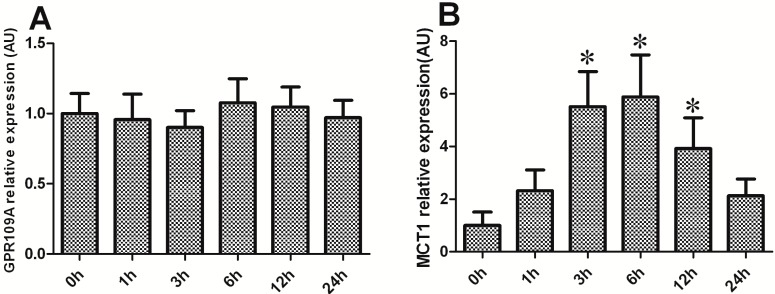
The effect of BHBA on the mRNA levels of *GPR109A* and *MCT1* in DCAPCs. **(A**) The effect of BHBA on the mRNA levels of *GPR109A* in DCAPCs; (**B**) The effect of BHBA on the mRNA levels of *MCT1* in DCAPCs. * indicates *p* < 0.05 *vs.* the control group.

GPR109A is the functional receptor for BHBA, and the uptake of BHBA into cells may be mediated by the monocarboxylate transporter 1 (MCT1) system. Thus, this study examined the effect of BHBA on the mRNA levels of *GPR109A* and *MCT1* in DCAPCs. *GPR109A* mRNA levels exhibited no obvious change in the BHBA-treated groups when compared with the control group (Figure 6A; *p* > 0.05). *MCT1* mRNA levels were significantly increased after BHBA treatment for 3, 6 and 12 h (Figure 6B; *p* < 0.05).

### 2.7. The Role of AMPK-α in BHBA-Regulated GH and PRL Transcription and Secretion

**Figure 7:**
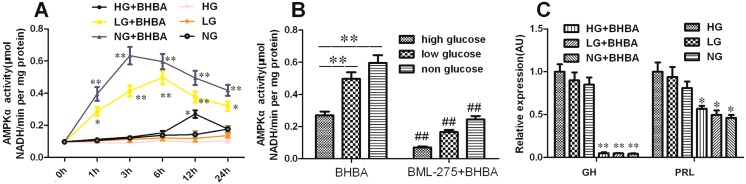

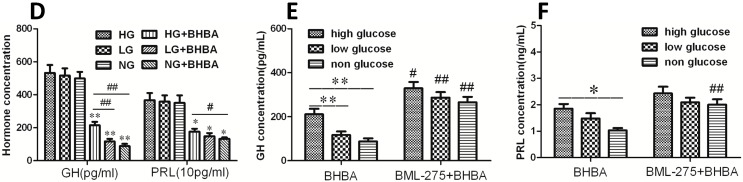
The role of AMPK-α in BHBA-regulated GH and PRL transcription and secretion in DCAPCs. (**A**) The effect of BHBA treatment on the activity of AMPKα in DCAPCs; (**B**) The effect of BML-275 treatment on the activity of AMPKα induced by BHBA in DCAPCs; (**C**) The effect of BHBA treatment on the gene expression of *GH* and *PRL* in DCAPCs; (**D**) The effect of BHBA treatment on the secretion level of GH and PRL in DCAPCs; (**E**) The effect of BML-275 treatment on GH secretion inhibited by BHBA in DCAPCs; (**F**) The effect of BML-275 treatment on PRL secretion inhibited by BHBA in DCAPCs. HG indicates high glucose (25.0 mM), LG indicates low glucose (5.5 mM), and NG indicates non-glucose (0.0 mM), respectively. * indicates *p* < 0.05 *vs.* the control group, ** indicates *p* < 0.01 *vs.* the control group, **^#^** indicates *p* < 0.05 *vs.* the prior PTX incubation group, ^##^ indicates *p* < 0.01 *vs.* the prior PTX incubation group.

The activity of AMPK-α displayed no obvious change in DCAPCs cultured in the low-glucose and non-glucose groups when compared with the high-glucose group (Figure 7A; *p* > 0.05). Incubation with 2.5 mmol/L BHBA resulted in a notable increase of AMPK-α activity in DCAPCs cultured in the low-glucose and non-glucose conditions when compared with the high-glucose group (Figure 7A). AMPK-α activity was markedly decreased by prior BML-275 treatment (Figure 7B; *p* < 0.01). For the DCAPCs cultured in the low-glucose and non-glucose conditions, incubation with 2.5 mmol/L BHBA had no obvious effect on *GH* and *PRL* expression compared with the high-glucose group (Figure 7C; *p* > 0.05). In contrast, the secretion levels of GH and PRL were notably decreased following BHBA treatment in DCAPCs cultured in the low-glucose and non-glucose conditions compared with the high-glucose group (Figure 7D). In addition, the trend toward decreasing secretion levels of GH and PRL was attenuated by prior BML-275 treatment (Figure 7E,F). These results suggest that the intracellular uptake of BHBA mediated by MCT1 may trigger AMPK signaling and result in decreases in *GH* and *PRL* mRNA translation.

## 3. Discussion

Growth hormone (GH) is a polypeptide hormone synthesized and secreted by the anterior pituitary gland. In lactating cows, GH induces the proliferation of mammary parenchyma and the growth of epithelial cells and increases cell renewal in the mammary gland [11]. Moreover, GH increases milk protein gene expression in bovine mammary explants and mammary epithelial cells [12,13]. PRL is a polypeptide hormone that is synthesized in and secreted from lactotrophs of the anterior pituitary gland [14]. PRL also play a key role in regulating mammary gland development and lactation [15]. Because of their integral regulatory role in growth, metabolism, and lactation, the factors and mechanisms affecting GH and PRL synthesis and release have been emphasized in endocrinology research.

BHBA has been identified as an endogenous ligand of GPR109A [5]. Plaisance *et al.* found that BHBA stimulates adiponectin secretion through its action on the GPR109A receptor [16]. GPR109A is a seven-transmembrane G protein-coupled receptor of the G_αi_ family that, in humans and mice, is expressed mainly in white adipocytes and immune cells such as monocytes and neutrophils.

In adipocytes, GPR109A activation results in the G_αi/o_ protein-mediated inhibition of adenylate cyclase, leading to a decreased cAMP response [17]. The suppression of cAMP has also been reported in GPR109A-transfected CHO-K1, 293EBNA, and HEK293 cells, which is ascribed at least in part to the inhibition of adenylate cyclase mediated by GPR109A [17,18,19]. In this study, we investigated whether BHBA mediate bovine *GH* and *PRL* gene transcription via the G protein signaling pathway. Our results demonstrate that BHBA can decrease intracellular cAMP concentration, PKA activity, and phosphorylation levels of CREB, by activating the G_αi_ subunit in DCAPCs. In the nucleus, phosphorylated CREB could either directly modulate *GH* gene transcription levels or indirectly activate Pit-1 to trigger the transcription of the *GH* and *PRL* genes [20,21,22]. In the present study, BHBA significantly down-regulated the phosphorylation levels of CREB. Thus, the expression and secretion of GH and PRL in DCAPCs were significantly decreased. PTX catalyzes the ADP-ribosylation of the α subunits of the heterotrimeric G_i/o_ protein family, thereby preventing the G proteins from interacting with their cognate GPCRs. This modification of the G_αi/o_ proteins results in the enhanced accumulation of cAMP, which is one of the mechanisms by which PTX induces the various biological effects in its host cells. Therefore, the expression and secretion levels of GH and PRL were significantly higher following prior PTX incubation. Based on these observations, we conclude that BHBA can inhibit *GH* and *PRL* gene transcription and secretion via the cAMP/PKA/CREB signaling pathway.

GPR109A belongs to a family of three GPCRs that share a significant sequence homology and whose known cognate ligands are metabolites of BHBA. GPR109A has generated increasing interest since its discovery as the receptor for niacin a decade ago. Taggart *et al.* demonstrated that the ketone body BHBA is a ligand for the receptor at physiologic concentrations [5]. Butyrate was also able to activate the receptor. As GPR109A’s primary pharmacological ligand in clinical use, niacin has been used for over 50 years in the treatment of cardiovascular disease, mainly due to its favorable effects on plasma lipoproteins [23]. In addition, the activation of GPR109A with agonists also mediates anti-inflammatory effects [23], tumor-suppressive effects [24], and immunoregulation [25]. Titgemeyer *et al.* first reported that cattle contain *GPR109A* in fat, liver, muscle, and brain tissue [6]. The authors suggested that the ability of BHBA to bind and activate GPR109A makes the unusual distribution of *GPR109A* in cattle especially intriguing. In this study, *GPR109A* mRNA was observed in bovine anterior pituitary gland, which suggests that BHBA may act directly at the level of the pituitary.

Cows can generate BHBA both in the liver by ketogenesis during a state of negative energy balance and via the oxidation of butyrate exclusive in ruminal epithelial cells [4]. In bovines, clinical ketosis is defined as increases in serum BHBA and NEFA concentration is association with a decrease in serum glucose concentrations [26]. Additionally, Zarrin *et al.* reported that plasma glucose concentrations decreased dramatically in response to an infusion of BHBA in lactating cows [27]. AMPK is activated under conditions that deplete cellular ATP and elevate AMP levels, such as occur during glucose deprivation and hypoxia [10]. In addition, the incubation of GT1-7 cells with BHBA in the 5.5 mM glucose medium were shown to modulate AMPK-α phosphorylation in GT1-7 cells [8]. Therefore, in the present study, we investigated whether BHBA regulates bovine pituitary function through the activation of AMPK-α signaling in cows during period of low blood glucose concentration. As expected, the levels of GH and PRL secretion were notably decreased after BHBA treatment in DCAPCs cultured in the low-glucose and non-glucose conditions in comparison with the high-glucose group. In addition, the observed trend of a decrease in levels of GH and PRL secretion was attenuated by prior BML-275 treatment. These results suggest that BHBA can trigger AMPK signaling and regulate bovine pituitary function.

AMPK is a heterotrimeric serine/threonine kinase that is involved in the maintenance of energy homeostasis and recovery from metabolic stress both at the cellular and whole-body level. Furthermore, AMPK has been found in all tissues examined thus far, and a number of its downstream targets have been identified. Several known AMPK substrates include key enzymes involved in lipid and glucose metabolism (e.g., Acetyl-coenzyme A carboxylase 1/2, ACC1/2; 3-hydroxy-3-methyl glutaryl coenzyme A reductase, HMG CoA; Insulin receptor substrate 1, IRS1), transcriptional components (e.g., CREB binding protein, CBP; Peroxisome proliferator-activated receptor γ, PPARγ), and components of the mammalian target of rapamycin (mTOR) signaling pathway (e.g., tuberous sclerosis complex, TSC1-TSC2) [28,29,30]. The CBP acts as a cofactor for the Pit-1-dependent activation of the hGH promoter by the GHRH signaling pathway and PKA [31]. Moreover, CBP acts by binding to phosphorylated CREB and activating gene expression [32]. The phosphorylated CREB may increase the levels of phosphorylated CBP or CBP complex, which interact with Pit-1 and result in the transcriptional activation of the *GH* and *PRL* genes. However, incubation with BHBA had no obvious effect on *GH* and *PRL* gene expression in DCAPCs cultured in the low-glucose and non-glucose groups. In contrast, GH and PRL secretion levels were notably decreased after BHBA treatment in DCAPCs cultured in the low-glucose and non-glucose groups. In mammals, the TSC1/TSC2-complex integrates environmental signals such as energy status and growth factors into mTOR signaling. In the case of stress (e.g., DNA damage, hypoxia) or low energy availability, the TSC1/TSC2-complex is activated and regulates protein synthesis down [33]. The trend toward decreasing secretion levels of GH and PRL was attenuated by prior BML-275 treatment. Thus, we hypothesize that BHBA may trigger AMPK signaling, and result in the phosphorylation of TSC1-TSC2, thereby leading to a reduction in *GH* and *PRL* mRNA translation via mTOR signaling. We found that the mRNA levels of *MCT1* were significantly increased after BHBA treatment. Based on these observations, we conclude that the intracellular uptake of BHBA mediated by MCT1 can trigger AMPK signaling and result in decrease in *GH* and *PRL* mRNA translation.

**Figure 8:**
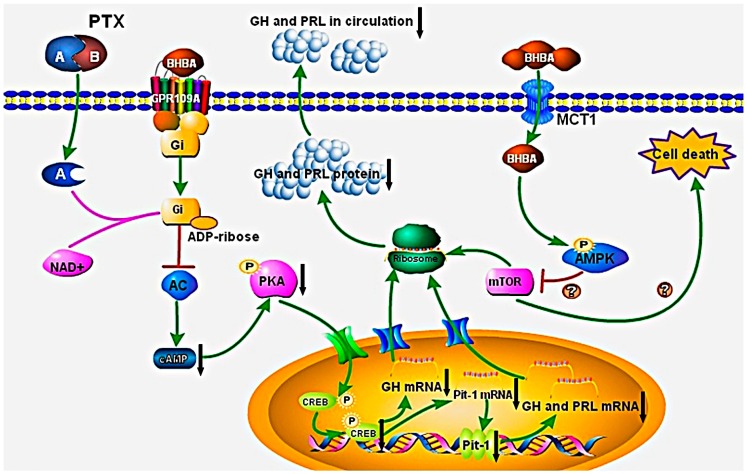
BHBA mediates GH and PRL gene transcription, translation, and secretion in DCAPCs via the cAMP/PKA/CREB and AMPK signaling pathways. BHBA binds to GPCR and leads to the dissociation of the heterotrimeric G protein complex into G_αi_ and G_βγ_ subunits. The exchange of GTP from GDP results in the activation of G_αi_, thereby inhibiting adenylyl cyclase (AC) activity. This process results in a decrease in intracellular cAMP levels and a subsequent reduction in PKA activity. The inhibition of PKA activity inhibits CREB phosphorylation, thereby decreasing GH and PRL gene transcription, translation, and secretion directly or indirectly. The A-protomer of PTX penetrates into the host cells and results in the inactivation of G_αi_, which subsequently inhibits the BHBA-mediated signaling pathway. In addition, intracellular BHBA uptake mediated by MCT1 may trigger AMPK signaling and result in the phosphorylation of TSC1-TSC2, leading to a decrease in *GH* and *PRL* mRNA translation via mTOR signaling.

In summary, the results of this study indicate that BHBA, acting as a signaling molecule, significantly decreases *GH* and *PRL* gene transcription in DCAPCs via the putative mechanism illustrated in Figure 8. Specifically, BHBA binds to GPR109A and leads to the dissociation of the heterotrimeric G protein complex into G_αi_ and βγ subunits. Next, the exchange of GTP from GDP results in the activation of the G_αi_, subunits, thereby inhibiting adenylyl cyclase activity. The inactivation adenylyl cyclase leads to a decrease in intracellular cAMP levels and a subsequent reduction in PKA activity. The inhibition of PKA activity inhibits CREB phosphorylation, which leads to a decrease in bovine *GH* and *Pit-1* gene transcription. The subsequent change in Pit-1 content results in the inhibition of transcription of the bovine *GH* and *PRL* genes. Consequently, BHBA inhibits bovine *GH* and *PRL* gene transcription and secretion in DCAPCs. The A-protomer of PTX penetrates into the host cells and results in the inactivation of G_αi_, thereby, inhibiting the BHBA-mediated signaling pathway. In addition, intracellular BHBA uptake mediated by MCT1 may trigger AMPK signaling and result in the phosphorylation of TSC1-TSC2, leading to a decrease in *GH* and *PRL* mRNA translation via mTOR signaling. mTOR integrates growth signals from diverse mechanisms that sense nutrient availability and as part of the response regulating cell survival [34]. We also found that cell survival was notably decreased after BHBA treatment in DCAPCs cultured under low-glucose and non-glucose condition when compared with the high-glucose group (our unpublished data). Therefore, BHBA may also decrease cell survival in DCAPCs cultured in condition of low and no glucose group. This study identifies a biochemical mechanism for the regulatory action of BHBA on *GH* and *PRL* gene transcription, translation, and secretion in DCAPCs, which may be one of the factors that regulate pituitary function during the transition period in dairy cows. Future studies are needed to clarify the specific role and mechanism of BHBA in regulating the survival of DCAPCs.

## 4. Materials and Methods

### 4.1. Isolation and Culture of DCAPCs

The cells were isolated and cultured by the enzymatic digestion method as previously described [35]. In brief, three Holstein cows (at the fifth lactational stage) anterior pituitary glands were diced into small pieces of less than 1 mm^3^, and incubated in Hanks’ balanced salt solution without calcium and magnesium (CMF–HBSS) containing 0.3% I type collagenase, 0.1% hyaluronidase and 0.1‰ DNase (Sigma, Shanghai, China) at 37 °C for 2 h. The dispersed cells were washed three times with HBSS and resuspended in Dulbecco’s modified Eagle’s medium (DMEM; Gibco, 25.0 mM glucose, Gibco, Carlsbad, CA, USA) supplemented with 10% fetal bovine serum (FBS; Gibco) at a seeding density of 1 × 10^3^ cells/mL. Then, the cells were seeded into a 75 cm^2^ culture flask (Corning, Tewksbury, MA, USA) and incubated at 37 °C in a humidified atmosphere containing 5% CO_2_. After 6 d in culture, the cells were treated with 0.5% trypsin (Sigma) and 0.02% EDTA in CMF–PBS and then seeded into 6-cm cell culture dishes (Corning). The anterior pituitary cells at the second passage were used to perform the experiment.

### 4.2. RNA Extraction, Reverse Transcriptase, and Quantitative Real-Time PCR Analysis

The time course of the experiments consisted of the following stages: DCAPCs were grown in 6-well plates (5 × 10^5^ cells), subsequently serum starved for 24 h, and then stimulated with 1.0 mmol/L dl-β-hydroxybutyric acid sodium salt (BHBA; Sigma) for 0, 1, 3, 6, 12, and 24 h. For the dose-response experiments, DCAPCs were stimulated with 0, 0.1, 0.5, 1.0, 2.5, and 5.0 mmol/L BHBA for 24 h. PTX is the ADP-ribosylating toxin produced by the whooping cough-causing bacterium Bordetella pertussis. ADP-ribosylation of the α subunit of heterotrimeric G_αi_ proteins locks the α subunits into an inactive state, thus preventing them from inhibiting adenylyl cyclase [36]. This method is widely applied as a tool in biochemical and pharmacological studies to investigate of signaling pathways involving heterotrimeric G proteins [37]. DCAPCs were also treated with or without prior PTX (Sigma) incubation (100 ng/L) for 2 h and then stimulated with 1.0 mmol/L BHBA for 24 h. Each treatment concentration of BHBA or PTX was replicated 12 times. The cell supernatants and cells were collected for hormone analysis and RNA extraction, respectively. Total RNA was isolated from cells with TRIzol reagent (Sigma) according to the manufacturer’s instructions and treated with DNase I (Takara, Kyoto, Japan) to prevent genomic DNA contamination. Total RNA concentration and purity were determined using a spectrophotometer (Bio-Rad, Hercules, CA, USA). Only samples with an optical density ratio at 260/280 nm in the range of 1.8–2.2 were used for further analysis. Total RNA integrity was checked by electrophoresis on an agarose gel. Total RNA samples were reverse transcribed using a reverse transcription kit (Takara) in accordance with to the manufacturer’s instructions. The expression levels of various genes were evaluated by quantitative polymerase chain reaction (qRT-PCR) analysis using the SYBR Green QuantiTect RT-PCR Kit (Roche, South San Francisco, CA, USA), performed in triplicate for each sample. The relative expression levels of bovine *GH*, *PRL*, *Pit-1*, *GPR109A* and *MCT1* (BHBA uptake via *MCT1*) genes were calculated relative to *GAPDH* (the normalizer) using the comparative cycle threshold method. The primer sequences for the tested genes are shown in Table 1 as previously described [6,38,39].

**Table 1:** The primer sequences of bovine *GAPDH*, *GH*, *PRL*, *Pit-1*, *GPR109A* and *MCT1*.

Gene	Sequences	Length (bp)
*GAPDH*	(F) 5'-TGCCCAGAATATCATCCC-3'	134
	(R) 5'-AGGTCAGATCCACAACAG-3'	
*GH*	(F) 5'-AGATCCTCAAGCAGACCTA-3'	121
	(R) 5'-AGGTACGTCTCCGTCTTA-3'	
*PRL*	(F) 5'-TATGAAAGGAGCCCCAGATG-3'	137
	(R) 5'-CACACAGGGTAGGGCTCAGT-3'	
*Pit-1*	(F) 5'-TTCTGCAACTCTGCCTCTGA-3'	148
	(R) 5'-CCATAGGTCGATGACTGGT-3'	
*GPR109A*	(F) 5'-ACATCACCCTCAGCTTCACC-3'	146
	(R) 5'-GCGGTTGTTATCCGACTCAT-3'	
*MCT1*	(F) 5'-GGAGTCATTGGAGGTCTTGG-3'	137
	(R) 5'-GCCAGGGTAGAGAGGAACAC-3'	

### 4.3. Measurement of GH and PRL Concentration in the Supernatant

GH and PRL in cell supernatants were evaluated with the corresponding ELISA kits according to the instructions of the manufacturer (Chemicon International, Millipore, Billerica, MA, USA).

### 4.4. Measurement of Intracellular cAMP Concentration

DCAPCs were grown in 6-well plates (5 × 10^5^ cells), serum starved for 24 h, and then stimulated with 1.0 mmol/L BHBA for 0.5, 1, 2, and 3 h. The intracellular cAMP was extracted from the cells and the concentration was assayed with a microplate reader (Bio-Rad, Hercules, CA, USA) using a cAMP assay kit (R&D Systems, Minneapolis, MN, USA) according to the manufacturer’s instructions. DCAPCs were also treated with or without prior PTX incubation (100 ng/L) for 2 h and then stimulated with 1.0 mmol/L BHBA for 3 h. Each treatment concentration of BHBA or PTX was replicated 12 times. The assay is based on the competition between unlabeled cAMP and a fixed quantity of horseradish peroxidase (HRP)-labeled cAMP for the limited number of binding sites on a cAMP specific antibody.

### 4.5. Measurement of PKA Activity

cAMP works by activating protein kinase A (PKA), which leads to the phosphorylation of cAMP response element binding protein (CREB). Thus, the PKA activity and phosphorylation state of CREB were determined in this study. DCAPCs were cultured in the presence or absence of BHBA (1.0 mmol/L) in serum-free medium for 3 h. The cells were also treated with prior PTX incubation (100 ng/L) for 2 h compared with groups without prior PTX incubation. Each treatment concentration of BHBA or PTX was replicated 12 times. The activity of PKA was determined by a radioactive method from a PKA assay kit according to the manufacturer’s instructions (Millipore).

### 4.6. Western Blotting Analysis of Phosphorylated CREB

Nuclear proteins were extracted using a nuclear protein extraction kit according to the manufacturer’s instructions (Beyotime Co., Nantong, China). Protein concentrations were determined with a bicinchoninic acid protein assay kit (Beyotime). Aliquots of cell lysates containing 20 μg protein were separated in 10% polyacrylamide gels and electrophoretically transferred to PVDF membranes (Millipore) using a Bio-Rad criterion blotter. Membranes were blocked in 5% non-fat dried milk in TBST at room temperature for 1 h and then incubated with either anti-phospho-CREB (1:3000 dilution; Millipore) or anti-CREB (1:2000 dilution; Millipore) at 4 °C overnight. The blots were then washed and incubated with horseradish peroxidase-labeled secondary antibodies (Sigma) at 37 °C for 1 h. Immunoreactive bands were detected with enhanced chemiluminescence Western blotting detection reagents (Beyotime). The blots were exposed to X-ray film for radiography of the bands and were digitally detected and measured using a LAS3000 Bioimage Analyzer (Fuji Photo Film, Tokyo, Japan).

### 4.7. Measurement of AMPK-α Activity

DCAPCs were grown in 6-well plates (5 × 10^5^ cells) and maintained in high-(25.0 mM), low-(5.5 mM), and non-(0.0 mM) glucose DMEM, serum starved for 24 h, and then stimulated with 1.0 mmol/L BHBA for 0, 1, 3, 6, 12, and 24 h. BML-275 is an AMPK-α inhibitor that inhibits AMPK-α phosphorylation at threonine-172. The cells were also treated with prior BML-275 incubation (10 μmol/L) for 2 h compared with groups without prior BML-275 incubation. Each treatment concentration of BHBA or BML-275 was replicated 12 times. Protein was extracted by cell lysis method described by Li *et al.* [40]. The enzyme activity of AMPK-α was detected using a biochemical kit via spectrophotometry (Shanghai Bluegene Biotech Co., Ltd., Shanghai, China) according to the manufacturer’s instructions.

### 4.8. Statistical Analyses

Results were expressed as means ± SD. Data were analyzed by using statistical software package SPSS 12.0 (SPSS Inc., Chicago, IL, USA). Groups were compared by one-way analysis of variance (ANOVA) followed by the least significant difference test. A *p* value of less than 0.05 was considered statistically significant, and values less than 0.01 were considered markedly significant.
